# Boosted Binary Quantum Classifier via Graphical Kernel

**DOI:** 10.3390/e25060870

**Published:** 2023-05-29

**Authors:** Yuan Li, Duan Huang

**Affiliations:** 1 School of Electronic Information Engineering, Shanghai Dianji University, Shanghai 200240, China; 2 School of Computer Sciences and Engineering, Central South University, Changsha 410083, China

**Keywords:** quantum computing, quantum classifier, nested graphical state, quantum entangle

## Abstract

In terms of the logical structure of data in machine learning (ML), we apply a novel graphical encoding method in quantum computing to build the mapping between feature space of sample data and two-level nested graph state that presents a kind of multi-partite entanglement state. By implementing swap-test circuit on the graphical training states, a binary quantum classifier to large-scale test states is effectively realized in this paper. In addition, for the error classification caused by noise, we further explored the subsequent processing scheme by adjusting the weights so that a strong classifier is formed and its accuracy is greatly boosted. In this paper, the proposed boosting algorithm demonstrates superiority in certain aspects as demonstrated via experimental investigation. This work further enriches the theoretical foundation of quantum graph theory and quantum machine learning, which may be exploited to assist the classification of massive-data networks by entangling subgraphs.

## 1. Introduction

Quantum computation is more effective at solving certain difficult problems than classical algorithms, and is considered as a significant part of theoretic physics [1,2,3]. Advances in quantum computing and machine learning naturally bring about the field of quantum machine learning in quantum information science [4,5,6,7,8,9,10,11,12]. Its emergence enriches and develops quantum information science and further bridges the two research areas. Classification of large-scale data is a significant application area of machine learning. The input data can achieve classification by learning from labeled data for pattern recognition. With regard to efficient quantum computing, the research of designing quantum classifier has attracted the attention of many researchers. Based on different data logical structures, some researchers have proposed distinct quantum classifiers, such as quantum support vector machine (SVM), decision tree classifiers and K-nearest neighbor (KNN) algorithms [4,5,6]. In general, a kernel measures similarity of data is applied to the design of distance-based and swap-test classification protocols in quantum mechanics.Graph state in quantum computation is a kind of essential multiparticle entangled state, which has been intensively researched with abundant results [13,14,15,16,17,18,19,20,21]. It represents well the structural and logical relationship between numerous quantum states.

In this paper, we establish the link between the so called two-level nested graph state and quantum sample state to reflect the structural characteristics of feature space. Based on the computational basis states, the large-scale data are encoded into graph state that is applied in quantum classifier with swap-test circuit. In terms of the generic kernel that is binary similarity measure, the test states may be classified resort to the fidelity probability. Due to the failed classification, some error samples will be further boosted through subsequent processing methods in our work. The remainder of this paper is arranged as follows. Firstly, we express the big-scale classical data in feature space with general quantum states and two-level nested graph states in Section 2. According to the efficiency of quantum graph states, a quantum classifier is proposed in the following section and the efficiency of measuring states is analyzed. Furthermore, in order to reduce the influence of noise, we adjust the attributes of test states to increase the fidelity of the quantum classifier. Experiments are carried out to illustrate the proposed algorithm by comparing the previous work and classical algorithms in Section 4. The conclusions are drawn in Section 5.

## 2. Quantum Machine Learning Based on Graphical Feature Space

As an example of pattern recognition in large-scale data analysis, a prominent application of machine learning is to predict the classification of input data categories by learning from labeled data. For classical machine learning, classification problem is an important subclass of supervised learning field. The computer model about classification is represented with labeled data and is required to learn some patterns. Therefore, most of the techniques in classical supervised machine learning are aimed at obtaining the best results by making use of the computational resources of polynomial quantities. We briefly describe the model of binary classification problems with quantum mechanism so that two labels are obtained by quantum measurement. Before designing the quantum classifier, we will first establish the relationship between the data logical structure and the feature space by encoding the quantum entangled state.

### 2.1. The Basic Map of Feature Space in Quantum Machine Learning

With classical machine learning algorithms, the labeled samples in set are needed to train a classifier. For binary classification, the input data can form a set of labeled feature vectors. Every sample is inherent in an *m*-dimension feature space $D_{m}$. Therefore, a sample vector in space $S_{N}$ is denoted as the tensor product of *N* input objets with their *m* features in terms of deferent attributes

(1) $$D_{m}\times S_{N}=D_{m}\times \overset{N}{\underset{n=1}{⨂}}S_{n}=D_{m}\times \left(S_{1}⊗S_{2}⊗\cdots ⊗S_{N}\right).$$

Assume a set of training sample dataset of inputs $X_{c}=\left\{x_{1},\ldots ,x_{N}\right\}\subset S_{N}$ which contains *N* samples, it may be described as

(2) $$E_{N}^{\prime }=\left\{\left(x_{1},y_{1}\right),\left(x_{2},y_{2}\right),\ldots ,\left(x_{N},y_{N}\right)\right\}\subset S_{N}\times \left\{0,1\right\}.$$

In the set, $x_{i}$ as a training sample attributed by *m* characters in another set $A_{c}=\left\{a_{1},a_{2},\ldots ,a_{m}\right\}$$\subset D_{m}$, with a designed classification algorithm, will be classified a class label $y_{i}\in Y=\left\{1,0\right\}$. The goal of the designed classifier is to classify an unseen datapoint *x* into *Y* as best as possible. In the following, we will consider their quantum expression with encoding method in graphical state.

Once the classical data are converted into quantum states through a certain coding method, quantum operation of the training and test data can be completed through a series of quantum operator gates [22]. The basic functions of the classifier are realized by using the projection measurement statistical information. By implementing the quantum circuit the designed classifier will be realized resort to the fidelity of quantum state overlap between training and test data [23]. In the preparation stage, input data are transformed to a quantum state which is described as the superposition of orthogonal eigenstates in subspace $S_{i}$. In high-dimensional Hilbert space $S_{N}$ space, every sample $x_{j}$ of *m*-dimension feature belongs to one of its orthogonal subspaces $S_{j}$.

Initially, the preparation of quantum sample state and test state that are evolved by unitary operator, is accomplished according to their encoding method with specific formats. A quantum state preparation circuit U:x→|x〉 is defined as a quantum feature map that acts on a vacuum state vector |0…0〉 in Hilbert space $S_{N}$. The whole quantum circuit may be realized by applying multi-qubit controlled operators with additional qubits. Single sample state $|x_{j}\rangle \in S_{j}$ is a superposition of *m* feature attributes in set $A=\{|a_{1}\rangle ,|a_{2}\rangle ,\ldots ,|a_{m}\rangle \}$. Accordingly, set $X=\{|x_{1}\rangle ,|x_{2}\rangle ,\cdots ,|x_{N}\rangle \}$ includes *N* quantum sample sates, of which elements are coded as form $\left(\sigma_{x}|\sigma_{z}\right)$ of Pauli operators. By implementing their quantum measurement, these sample states will be labeled in set *Y* resort to the results [24]. Hence, the quantum training dataset of containing *N* sample states with their labels can be indicated as

(3) $$E_{N}=\left\{(|x_{1}\rangle ,y_{1}\right),(|x_{2}\rangle ,y_{2}),\cdots ,(|x_{N}\rangle ,y_{N})\}.$$

**Figure 1:**
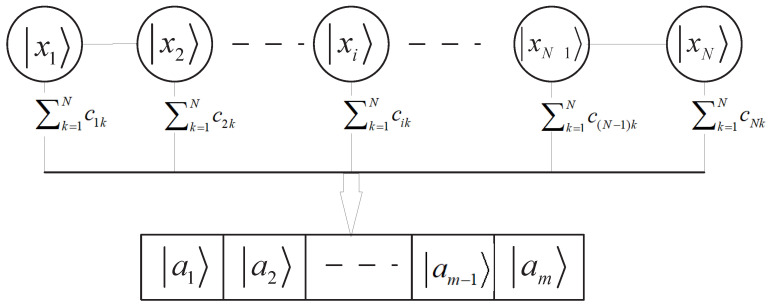
Expression of the inputting quantum state. Here, every $|x_{j}\rangle$ in subspace $S_{j}$ is a quantum sample state in subspace $S_{j}$ described by the superposition of orthogonal eigenstates $|a_{k}\rangle$. Furthermore, $c_{jk}$ are corresponding coefficients for 1≤j≤N and 1≤k≤m.

According to the above definitions, any quantum sample state $|x_{j}\rangle$ in subspace $S_{j}$ can be expressed as a superposition of orthogonal eigenstates, i.e., $|x_{j}\rangle =\sum_{k=1}^{m}c_{jk}|a_{k}\rangle$, where the complex number $c_{jk}$ is probability amplitude of feature state $|a_{k}\rangle$ for $\sum_{k=1}^{m}{\left|c_{jk}\right|}^{2}=1$. The construction relationship of training state and feature states may be depicted as seen in Figure 1.

Generally, a designed quantum classification algorithm should refer to the state discrimination or detection rather than pattern classification. The goal of designing a quantum classifier is to exploit quantum effects to achieve data classification that surpasses the classical one in terms of the computational complexity [24,25].

### 2.2. Two-Level Quantum Nested Graphical States Mapped to Feature Space

In practical applications, there are large-scale data involved in machine learning. Hence, it is vital to prepare the big classical data as quantum state with some coding mode [26]. In the quantum-information-processing task, multiparticle entanglement is a critical part because its controlled generation has been proved in many physical systems [27,28,29]. For expressing the classical data in quantum machine learning as the corresponding quantum version by coding process, graph state may be more proper because of its entanglement character. To design a quantum classifier, we firstly map classical large-scale samples and their features for efficient measurement to the entangled graph state.

A graph is composed of a set of vertices and edges describing the connection of vertices. Here, vertex indicates datapoint in the graph, and edge represents the interaction between these vertices, respectively [14,17,19].

**Definition** **1.** *Typically, an undirected and finite graph in data logic structure may be described as form G=(V,E). Here, V is a set of vertices that contains the input sample states, and E is the set of edges that establishes the entanglement relation between vertices. Furthermore, N=|V| is the sample state number of graph state |G〉. Vertices a,b∈V are two endpoints of an edge, which are called adjacent. An N×N adjacency matrix* Γ *in the graph G describes the symmetric connection relation between all sample states in set V, of which elements may be denoted as*

(4) $$\begin{array}{c} \Gamma_{a,b}=\left\{\begin{array}{cc} 1 & \mathrm{if}(a,b)\in E\\ 0 & \mathrm{otherwise} \end{array}\right.. \end{array}$$

In addition, another matrix known as a generator matrix of the graph state may be expressed as binary form $G=\left(\sigma_{x}|\sigma_{z}\right)=\left(I|\Gamma \right)$ for identity matrix *I* and adjacency matrix Γ. Furthermore, in terms of mapping $\sigma_{x}\to x$, the generator matrix of above form also may be expressed as C=Γ+ωI over $GF\left(4\right)=\left\{0,1,\omega ,\omega^{2}\right\}$. Every vertex in entangling *N* sample states corresponds uniquely to an *N*-qubit vertices vector. Thus, based on the above definitions, we map the *N*-sample set to the vertex set $V_{N}$ in subgraph $G_{N}$, and feature space $D_{m}$ to another subgraph $G_{m}$ with *m* vertices, i.e., there is map *f* that can link the space

(5) $$f:\;\;D_{m}\times S_{N}\mapsto G_{m}\times G_{N}.$$

**Figure 2:**
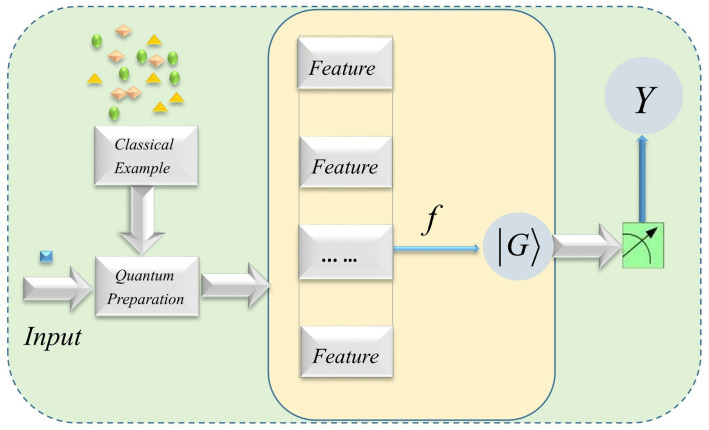
Illustration of using quantum feature maps based on graph state *G* for machine learning. According to the existing samples, the input data describing different shapes can be classified into the labels in *Y*. Here, the different shapes depicted in yellow, purple and green are different classes of classical samples, as well as the blue square represents the input data to be classified.

The physical circuit of whole classification process can be described as Figure 2.

To build the relation between the two subgraphs based on the data structure in machine learning, we apply construction method of nested graph as follows.

**Definition** **2.** *A nested graph G of containing $N_{g}$ states called as an “$n_{l}$-vertices graph of ⋯ of $n_{2}$-vertices graph of $n_{1}$-vertices graph” [30], is denoted by $G_{n_{l}}\left[\cdots \left[G_{n_{2}}\left[G_{n_{1}}\right]\right]\right]$. Here, we call it as l-level nested graph. The total vertex number of corresponding to states in the graph is $N_{g}=n_{1}n_{2}\cdots n_{l}$, and the obtained graph G is formed by the $N_{g}/n_{1}$ disjoint vertex subsets $V_{1},V_{2},\cdots ,V_{N_{g}/n_{1}}$ of same size $n_{1}$. Here, set $V_{i}$ (1≤i≤l) is the vertex sate dataset of subgraph $G_{n_{1}}$. This method practically is a way of employing tree structure to generate quantum states of massive sample data.*

According to structural characters of sample state, for further expressing the *N* sample states and their *m* attributes to every one, we shall consider two-level nested graph state

(6) $$G=\left[G_{n_{2}}\left[G_{n_{1}}\right]\right]=\left[G_{N}\left[G_{m}\right]\right],$$

with two subgraphs $G_{n_{1}}=G_{m}$ and $G_{n_{2}}=G_{N}$, of which vertex number is $N_{g}=Nm$. Here, the entanglement relationship of sample states may be depicted by graph state $|G_{n_{1}}\rangle$, and their *m* feature states may be expressed by vertices in graph state $|G_{n_{2}}\rangle$. In the coding process, another problem is how to provide a general method of obtaining its adjacent matrix in constructing nested graphical quantum codes. To reduce the influence of errors and decoherence, the coding method may be chosen as low-density parity-check (LDPC) codes which possess good performance for encoding massive data and corresponds to a sparse graph with low complexity of decoding. In light of the above definition, we connect the subgraphs of two-level nested graph state |G〉 with adjacent matrix $\Gamma_{G}$ to gain its generator matrix. For obtaining large amounts of data in machine learning, assume the two-level nested graph *G* is entangled by the two disconnected subgraphs $G_{n_{1}}=\left(V_{n_{1}},E_{n_{1}}\right)$ and $G_{n_{2}}=\left(V_{n_{2}},E_{n_{2}}\right)$ with respect to entanglement relation matrix $\Gamma_{n_{12}}$. Here, we denote $V_{n_{1}}$ as the set of sample states, and $V_{n_{2}}$ as their feature set, and $E_{n_{1}}$ and $E_{n_{2}}$ are their edge set respectively. After constructed subgraphs $G_{n_{1}}$ and $G_{n_{2}}$, denote $\Gamma_{n_{2}}$ and $\Gamma_{n_{1}}$ as the adjacent matrices of the two subgraphs, respectively, which depict the relationships of *N* sample states and *d* feature states, respectively [31].

To entangle the two subgraphs, we consider a cyclic group generated in terms of the following rules. For an integer *L*, a circulant permutation matrix $P_{L}=\left(p_{ij}\right)$ of which elements are defined by

(7) $$\begin{array}{c} p_{ij}=\left\{\begin{array}{cc} 1 & \mathrm{if}i=(j+1)\mathrm{mod}L\\ 0 & \mathrm{otherwise} \end{array}\right.. \end{array}$$

Then, a finite group with $P_{L}$ is formed as

(8) $$\begin{array}{c} T_{L}=\left\{P_{L}^{0}=I,P_{L},\ldots ,P_{L}^{L-1}\right\}. \end{array}$$

**Figure 3:**
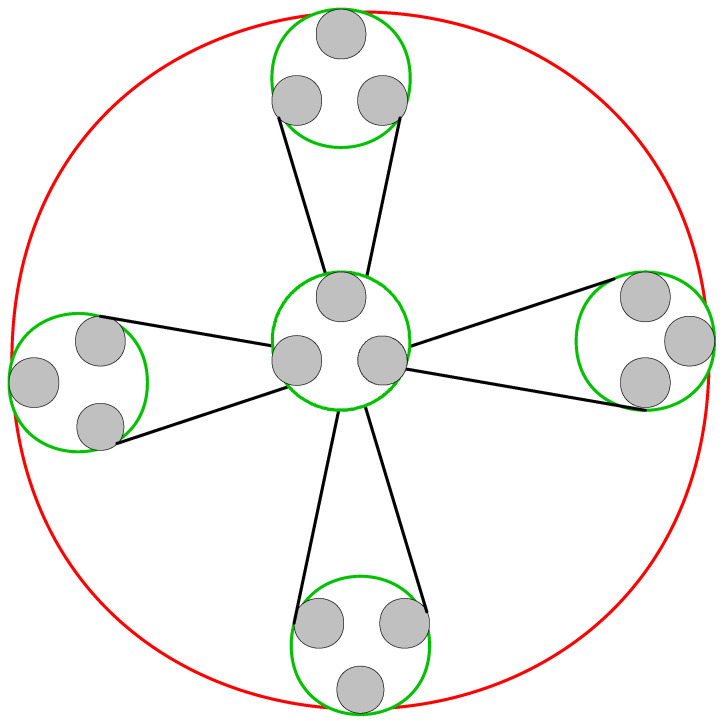
A two-level nested graph $G=[G_{5}\left[G_{3}\right]]$ which corresponds to graph state |G〉. Here, the subgraph $G_{3}$ is formed by three grey entangling dots, and graph *G* entangled by the five subgraphs.

Any element in set (8) may be used to generate the simple entanglement matrix about the entanglement relationships of sample states and feature states. For example, a two-level nested graph $G=[G_{5}\left[G_{3}\right]$ is shown in Figure 3, in which graph *G* is generated by entangling 3-feature subgraph $G_{m}=G_{3}$ and 5-sample subgraph $G_{N}=G_{5}$.

If the above described subgraphs correspond to two graph states, the entangled graph also corresponds to a stabilizer state. Therefore, in terms of the circulant permutation matrix (7), a stabilizer generator matrix of length *N* which corresponds to the two-level nested clique graph *G* in (6) will be obtained. After obtaining a set of independent generator matrices, in terms of following Singleton bound

(9) N≥k+d−1,

a graphical quantum code [[N,k,d]] may be generated by selecting N−k row sample vectors from the *N*-level concatenated matrix as the generators [32]. In fact, a stabilizer can be specified with the matrices by taking an N−k-dimension subspace of $S_{N}$ on quantum code [[N,k,d]] [33,34].

## 3. Swap-Test Quantum Classifier with Large-Scale Data

On the basis of the quantum graph states encoded before, a quantum classifier is designed to obtain classification function $H(\vec{x})$ in the following, of which the main principle is to regard the entire classification process as the evolutionary process of a closed quantum system. In fact, the evolution is achieved with a series of quantum logic gates on the graph state, and an *N*-site lattice with a qubit is attached to each site.

### 3.1. Quantum Swap-Test Classification Based on Graph State

At present, distance-based quantum classifier and the swap-test classifier are usually implemented to design quantum classifiers [8,29]. Resort to an entangled two-level graph state |G〉 described previously, given *M* obtained unseen states $|{\hat{x}}_{1}\rangle ,|{\hat{x}}_{2}\rangle ,\ldots ,|{\hat{x}}_{M}\rangle$ are to be classified simultaneously and regarded as the query states of which any one mapped by the feature space expanded into space $S_{N}$.

To realize the process of the whole quantum classifier classifying samples, it is necessary to first prepare the test state and the training state. In general, the probability amplitudes of the training states and test states will be initially equal after completing entanglement in two graphs. Any training states of *N* two-level nested graph state $|G_{t}\rangle$ of which vertices are indicated $|\varphi_{1}\rangle ,|\varphi_{2}\rangle ,\ldots ,|\varphi_{N}\rangle \in S_{N}$, are constructed as

(10) $$|\varphi_{j}\rangle =1/\sqrt{N_{j}}\cdot \sum_{n=1}^{N}x_{j,n}\left|n\rangle \right|x_{n}\rangle$$

for $N_{j}=\sum_{n=1}^{N}{\left|x_{j,n}\right|}^{2}$. Based on its feature space $D_{m}$, the training state further can be described as

(11) $$\begin{array}{c} |\varphi_{j}\rangle =\frac{1}{\sqrt{N_{j}}}\sum_{n=1}^{N}\sum_{k=1}^{m}c_{jk}x_{j,n}|a_{k}\rangle |n\rangle . \end{array}$$

Here, state $|a_{k}\rangle \in A$ describes *k*th feature of quantum sample state $|x_{j}\rangle \in X$. Practically, to classify *M* query state vector $|\vec{x}\rangle =\left({\hat{x}}_{1},{\hat{x}}_{2},\ldots ,{\hat{x}}_{M}\right)$ which is entangled as graph state $|G_{q}\rangle$ on the basis of sample states, we consider classifiers $h_{1}\left(\hat{x}\right),h_{2}\left(\hat{x}\right),\ldots ,h_{M}\left(\hat{x}\right)$ to obtain the error rates of query states. We construct the oracle state

(12) $$|\psi_{j}\rangle =1/\sqrt{{\hat{N}}_{j}}\sum_{n=1}^{N}{\hat{q}}_{jn}|{\hat{x}}_{j}\rangle |n\rangle ,$$

for ${\hat{N}}_{j}=\sum_{n=1}^{N}{\left|{\hat{q}}_{jn}\right|}^{2}$ and ${\hat{q}}_{jn}=c_{jk}x_{j,n}$, that contains any query state $|{\hat{x}}_{j}\rangle$ which will be classified by quantum classifier $h_{j}$. For the classification of *M* query states, based on the constructed states $|\varphi_{j}\rangle$ and $|\psi_{j}\rangle$, an ancilla state is firstly prepared in a swap-test classifier as

(13) $$\begin{array}{c} |\phi_{j}\rangle =\frac{1}{\sqrt{2}}(|\varphi_{j}\rangle \left|0\rangle +\right|\psi_{j}\rangle |1\rangle )=\frac{1}{\sqrt{2}}(\frac{1}{\sqrt{N_{j}}}\sum_{n=1}^{N}|x_{n}\rangle \\ |n\rangle +\frac{1}{\sqrt{{\hat{N}}_{j}}}\sum_{n=1}^{N}{\hat{q}}_{jn}|{\hat{x}}_{j}\rangle |1\rangle )|n\rangle =\frac{1}{\sqrt{2}}(\frac{1}{\sqrt{N_{j}}}\sum_{n=1}^{N}\sum_{k=1}^{m}\\ c_{jk}|a_{k}\rangle |0\rangle +\frac{1}{\sqrt{{\hat{N}}_{j}}}\sum_{n=1}^{N}{\hat{q}}_{jn}|{\hat{x}}_{j}\rangle |1\rangle )|n\rangle . \end{array}$$

To obtain the classification result by measuring the ancilla state $|\phi_{j}\rangle$, another state

(14) $$|\phi_{j}^{\prime }\rangle =\frac{1}{\sqrt{2}}(|0\rangle -\left|1\rangle \right)$$

will be used in quantum computation. On the one hand, without regard to the entanglement, the *M* training and query states are prepared as

(15) $$\begin{array}{c} |\Phi \rangle =\frac{1}{\sqrt{2}}\left(I^{⊗M}(\sum_{i=1}^{N}\frac{1}{\sqrt{N_{\varphi }}}\lambda_{i}|\varphi_{j}\rangle \right)|0\rangle +\prod_{j=1}^{M}|\psi_{j}\rangle |1\rangle ), \end{array}$$

where $\lambda_{i}$ is the attribute of state $|\varphi_{j}\rangle$ for $N_{\varphi }=\sum_{i=1}^{N}{\left|\lambda_{i}\right|}^{2}$. The running time of the inner product of two states $|\phi_{j}\rangle$ and $|\phi_{j}^{\prime }\rangle$ to achieve the success is O(M). On the other hand, if entanglement property is considered, each quantum state $|\varphi_{j}\rangle$ can be regarded as a two-level nested subgraph. By entangling *M* subgraphs with direct product relationship, the quantum state $|\Phi_{j}\rangle$ that likes form (15) will be transformed into a graph state $|G_{t}\rangle$. In light of the entanglement method of subgraphs, circulant permutation matrix in (7) can be used as an adjacency matrix. Similarly, the *M* query states and training states can be entangled in graph states $|G_{q}\rangle$ and $|G_{t}\rangle$, respectively, hence the corresponding state prepared as

(16) $$\begin{array}{c} |\Phi^{g}\rangle =\frac{1}{\sqrt{2}}(|G_{t}\rangle \left|0\rangle +\right|G_{q}\rangle |1\rangle ). \end{array}$$

On the basis of *M* query states, the running times of entanglement method is O(M).

The performed accuracy of measuring classification can be obtained by $p_{j}={\left|\langle \phi_{j}|\phi_{j}^{\prime }\rangle \right|}^{2}$. Furthermore, it is indicated by the two states containing the training state and test state as $\frac{1}{2}\left(1-\langle \varphi_{j}|\psi_{j}\rangle \right)$. Denote $\kappa_{j}=\langle \varphi_{j}|\psi_{j}\rangle$, which also can be described as

(17) $$\kappa_{j}=\frac{1}{\sqrt{N_{\kappa_{j}}}}\sum_{n=1}^{N}\sum_{k=1}^{m}{\hat{q}}_{jn}c_{jk}x_{jn}\left|{\hat{x}}_{j}\right|\langle x_{n}|{\tilde{x}}_{j}\rangle .$$

where $\sqrt{N_{\kappa_{j}}}=\sum_{n=1}^{N}\sum_{k=1}^{m}{\left|r_{j,nk}\right|}^{2}$ and $r_{j,nk}={\hat{q}}_{jn}c_{jk}x_{jn}$. When the parameter $\kappa_{j}$ ranges from $0<\kappa_{j}<1$, i.e., $p_{j}<1/2$, the query state $|{\hat{x}}_{j}\rangle$ is classified as 1, otherwise 0. Therefore, only parameter $\kappa_{j}$ meets the requirement, a classifier $h_{j}\left({\hat{x}}_{j}\right)$ can be obtained for sample state $|\vec{x}\rangle$. A mixed-state diagonal based on the graph-state basis can correspondingly be described as the following form ρ=|Φ〉〈Φ| or $\rho^{g}=|\Phi^{g}\rangle \langle \Phi^{g}|$ according to whether it is entangled, of which diagonal elements can be obtained through calculation as the result $p_{j}$(j=1,2,…,N). Therefore, the gained vector of success probability $\vec{p}=\left[p_{1},p_{2},\ldots ,p_{M}\right]$ which distinguishes from the sample states may correspondingly be obtained.

Based on the above classification process of single query state, a vector *H* containing *M* classifiers $h_{1},h_{2},\ldots h_{M}$ can be obtained, regardless of whether the *M* test states are entangled or not. The superposition state vector of graph state may be shown as $H\left({\hat{\vec{x}}}_{M}\right)=\sum_{n=1}^{M}r_{n}h_{n}\left({\hat{x}}_{n}\right)$, where $\sum_{n=1}^{N}r_{n}=1$ for weights $r_{n}$ and ${\hat{\vec{x}}}_{M}$ is test state vector. The arbitrary probability amplitude also can be uniformly weighted with equal probability amplitude, i.e., $r_{n}=1/M$. Since the fork states in the classification process may be altered by multiplicative and additive noise, the ultimate outcome will be changed. Denote $\varepsilon_{j}$ as the error rate of classifying query state ${\hat{x}}_{j}$, which is close to the parameter $\kappa_{j}$. The occurrence of the two probabilities obeys the same probability distribution. The error dependence to the test state in training stage is *O*(poly(M/(1−ε))).

### 3.2. Quantum Graph Kernels and Graph Segmentation

**Figure 4:**
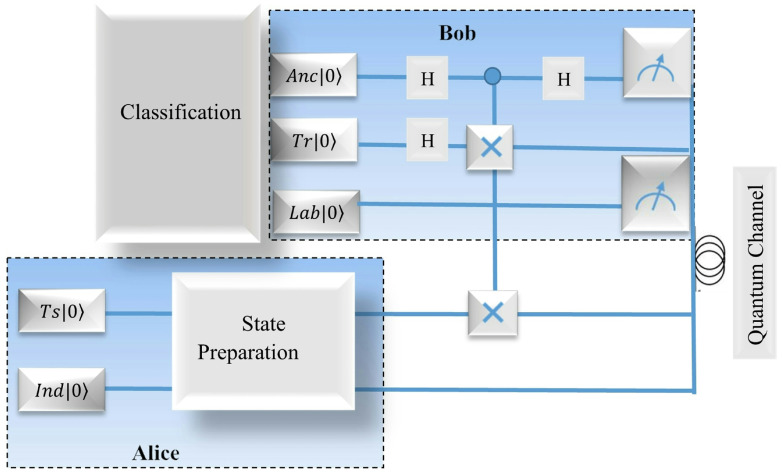
The circuit of classifier in quantum communication for two correspondents (Alice and Bob). The test state is prepared with operator ‘Ts’ at Alice’s side, of which features are indexed by ‘Ind’. By entangling an ancilla state operated by operator ‘Anc’ with the training state gained by ‘Tr’ at receiver Bob’s side. As a result, the class label may be derived from ‘Lab’ after measuring.

In practice, the classifier may be used in quantum communication to classify the test data from sender, and to label them at the receiver with training data in quantum field. Assume Alice and Bob are two sides of the communication party, the experimental setup of the quantum classifier can be shown in Figure 4.

In the following, we will briefly analyze the empirical results of a distance-based quantum classifier implemented on five-qubit quantum processor in this figure. Firstly, we encode the classical data point into amplitudes of quantum graph states. In terms of the transformation, the quantum state fidelity may be achieved by method of swap-test. Specifically, we employ the Hadamard gate to the data qubit containing a test point and a training point in the sample set. According to the designed algorithm, the superposition and entanglement are exploited in physical circuit to evaluate the distance between the two points. During the preparation stage of the sender’s side, Alice prepares a test state Ts|0〉 as the input data, which possesses *m* characters indexed by state Ind|0〉. At Bob’s side, he applies two Hadamard gates on superposition of an ancilla qubit Anc|0〉 and a training state Tr|0〉. Furthermore, another Hadamard gate is employed among the controlled gates by pitching in the qubits to achieve the entanglement. As a result, the quantum circuit at Bob’s side can classify its class Lab|0〉 in label $y_{n}$ in set *Y* which the test point belongs to. As can be seen from the typical quantum circuit, it includes the preparation stage of initializing the graph states at Alice’s side by making use of the unitary operators, and the measurement stage of obtaining the measurement results at Bob’s side.

The rapid development of quantum computing has greatly promoted the research of graph representation which maps graph into vector space to facilitate various downstream works [35]. Generally, quantum graph representation algorithm bears significant capabilities in extracting some atypical patterns in graphs [36]. In particular, quantum graph *G* can effectively reflect the data structure of quantum states, such that the features of graph are characterized by the entanglement and superposition. Generally, the two representative methods of graphical quantum machine learning are quantum graph segmentation and graph kernel.

With the development of quantum devices, the massive data in graph *G* can be segmented into *s* subgraph $G_{1},G_{2},\ldots ,G_{s}$ for $G={⋃}_{j=1}^{s}G_{j}$, where *s* is an integer. Therefore, to classify the large-scale data in graphical network with a classifier $H_{g}$ can be combined by a family of corresponding classifiers $H_{1},H_{2},\ldots ,H_{s}$ involved in its subgraphs, respectively, i.e., $H_{g}=\sum \tau_{i}H_{i}$ and $\sum_{i=1}^{s}\tau_{i}=1$ for the weight coefficients $\tau_{i}$ (1≤i≤s) which is generally initialized as 1/s. Assume $\Gamma_{i,j}$ defined in Equation (4) is the adjacent matrix (graph) between any two subgraphs $G_{i}$ and $G_{j}$ therein. As a result, a quantum graph neural network can be formed, hence fault-tolerant computer-based quantum algorithms can be applied to accelerate the calculation efficiency of classical models.

On the other hand, to distinguish the differences between two graph states, the kernel is defined. Without loss of generality, we consider the case of two graphs $G_{1}$ and $G_{2}$ in Equation (16) with their adjacent matrix (graph) $\Gamma_{1,2}$. The input data can be generally encoded into quantum amplitudes with sparse graph that has the low-density performance. In its feature space, quantum graph kernels represent different graphs and compare their similarity with the inner product between them [37]. Assume C is a low-density encoder of mapping two graphs $G_{1}$ and $G_{2}$ into quantum state in a Hilbert space, their similarity can be measured by a kernel *K* of the two graphs

(18) $$\begin{array}{c} K\left(G_{1},G_{2}\right)=\langle C\left(G_{1}\right)|C\left(G_{2}\right)\rangle . \end{array}$$

**Figure 5:**
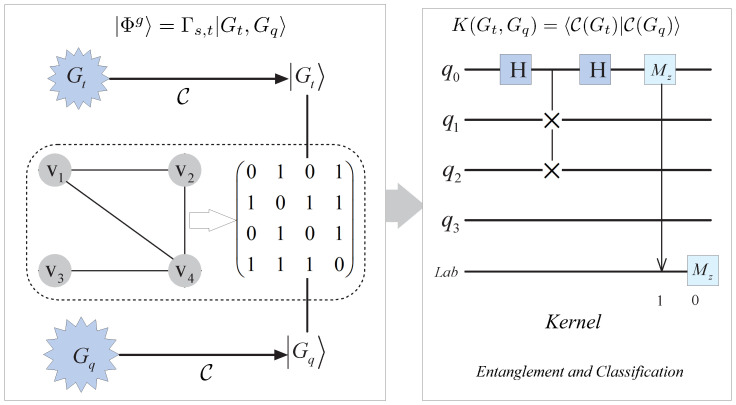
Illustration of quantum graph segmentation and graphical kernel. A graph state $|\Phi^{g}\rangle$ is formed by graphs $G_{t}$ and $G_{q}$ which correspond to training state $|G_{t}\rangle$ and query state $|G_{q}\rangle$ with a mapping C, where the two states are entangled by entanglement matrix (graph) $\Gamma_{s,t}$. Here, $v_{i}$ for 1≤i≤4 in the circuit are the vertices in graphs $G_{t}$ and $G_{q}$. In the circuit of kernel, the first register is the ancilla state, and the second is the training state. Furthermore, the third register is the input data as the query state. Correspondingly, the fourth register and final register are label and index states, respectively, so that the label results may be obtained with phase measurement $M_{z}$.

Assume a graph state $|\Phi^{g}\rangle$ in Equation (16) is entangled by two graph states $|G_{t}\rangle$ and $|G_{q}\rangle$ which correspond to two graphs $G_{t}$ and $G_{q}$ with mapping C. In order to take further advantage of the kernel based on the quantum graph state, rather than the only real part of quantum state overlap as introduced in ref. [29], we consider the quantum classifier based on the graph state. The whole quantum circuit is shown in Figure 4. Here, it takes the nodes in the the two graphs with their entanglement matrix (graph) $\Gamma_{t,q}$, such as the entangled vertices $v_{i}$ for 1≤i≤4 in Figure 5.

In this figure, there are five registers based on the graphical kernel in the quantum circuit. To distinguish the difference between the graph states $|G_{t}\rangle$ and $|G_{q}\rangle$, an ancilla qubit is prepared in first register. State $|x_{n}\rangle$ in $G_{t}$ as the training state formed from state in Equation (10) in the proposed method is stored in the second register, and any state $|\hat{x}\rangle$ among *M* query states in graph $G_{q}$ is in the third. Correspondingly, the label qubit |y〉 and index |n〉 in the Equation (11) correspond to the fourth and final registers, respectively. As a result, its label will be obtained with phase measurement $M_{z}$. The process of swap-test may be depicted in the following form

(19) $$\begin{array}{c} \sum_{n=1}^{N}\sqrt{w_{n}}\left|0\rangle \right|x_{n}\rangle |x\rangle |y_{n}\rangle |n\rangle \to |\Phi^{g}\rangle \end{array}$$

where $w_{n}$ is the weight for $\sum_{n=1}^{N}w_{n}=1$. Then, the classification result will be obtained by the measure operators.

### 3.3. Fidelity Analysis in Quantum Classifiers

In the quantum circuits in Figure 4, some test states are erroneously measured with ancilla qubits, hence heir class labels may be incorrect. To boost the fidelity, a soft algorithm is explored by Bob, which will be introduced in a later subsection. To test the binary proposed classifier depicted in the quantum circuit in the two figures, in terms of the proposed algorithm, classical input data $\hat{x}$ for binary classification can be encoded into amplitude and normalized as following form among [−π,π]

(20) $$|\hat{x}\rangle =\sin\theta |0\rangle +\cos\theta |1\rangle .$$

**Figure 6:**
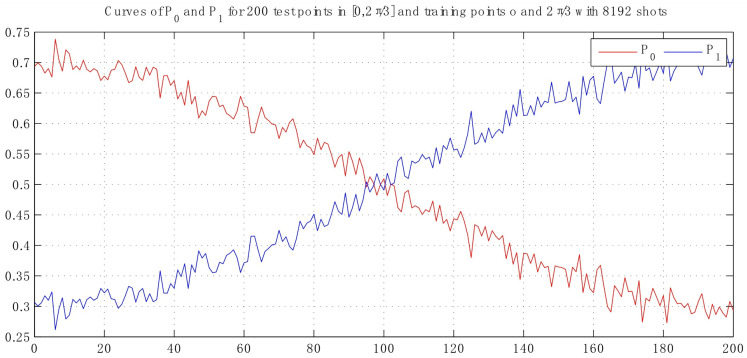
Curves of probability with 200 test points in interval [0, 2π/3] resort to 8192 shots, which varies in the range [0.26, 0.74].

This means that the data may be encoded with pair (sinθ,cosθ) for parameter θ. Denote the probabilities $P_{0}$ and $P_{1}$ of classifying input state as the binary labels 0 and 1, respectively, for $P_{0}+P_{1}=1$. For simplicity, we take θ to represent the input test point, that can be labeled as classes 0 and 1 with amplitude 0 and θ, respectively. Thus, the test point is selected uniformly in the interval [0, θ] as the query point. In following experiment, angular parameters θ=2/3π and θ=1/6π are taken. In Figure 6, the interval is [0, 2/3π] that the class label can be basically distinguished with 8192 shots.

With the same quantity of shots shown in Figure 4, the result becomes indistinct so that it can not be distinguished clearly while the angular parameter θ=1/6π is chosen.

Therefore, it is obvious that the angular distance of the two training points become smaller, so the fidelity will be reduced.

**Figure 7:**
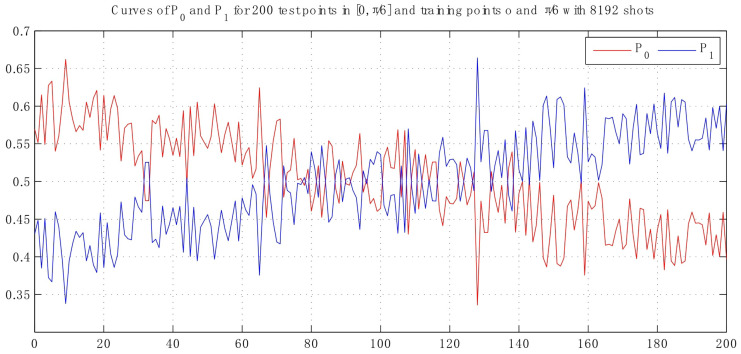
Curves of probability with 200 test points in interval [0, π/6] resort to 8192 shots, which varies in the range [0.34, 0.66]. Here, we take the smaller angular distance.

**Figure 8:**
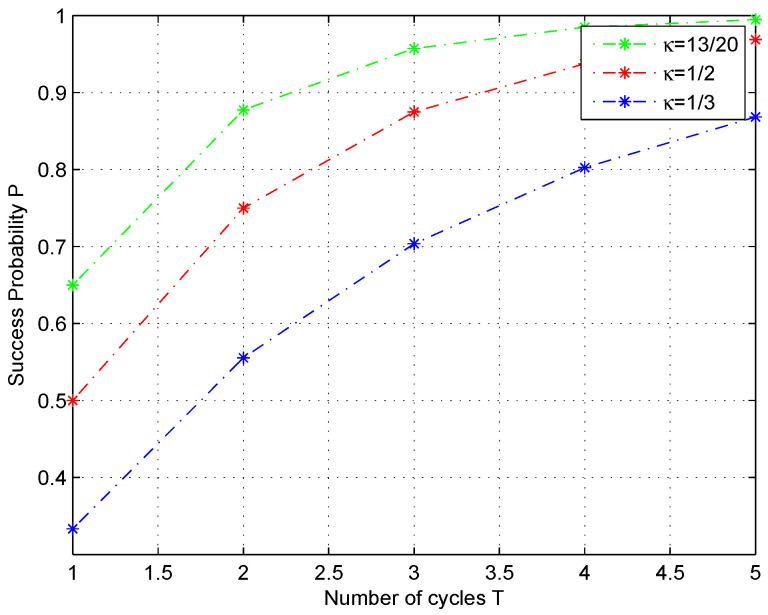
Convergence trend after adopting iterative cycles. The probability *p* of classification while the parameter κ is taken as κ=12/20,1/2,1/3, respectively, in the quantum classifier, the accuracy approaches 1 with different velocities in terms of the number *T* of adopted iterations.

By comparing with traditional classification algorithms, the improved algorithm can reduce the weights of correctly classified samples and allow the base classifier to pay closer attention to difficult to distinguish samples. Therefore, it results in the higher classification accuracy of the proposed method. As observed in Figure 6 and Figure 7, the fidelity will decrease when their angular distance reduces, so that the classification task becomes harder. Due to the large amount of data involved in the classification process, there are inevitably some test states that will be classified incorrectly. We can see that the failed classification parameter $\kappa_{i}$ is around 1/2. Given that the threshold is $\kappa_{0}$, it satisfies $|\kappa_{i}-1/2|<\kappa_{0}$. Assume the iteration time number is denoted as *T*, if the threshold condition is still not met and the weight is not considered, the probability will be $1-{\left(1-\kappa_{i}\right)}^{T}$ under the fact that each iteration is independent. As a result, the accuracy will be promoted with each subsequent iteration, as can be illustrated in Figure 8.

This shows that the success probability *p* is close to 1 under an iterative cycle of T=5 while the parameter κ is taken as different values around 1/2. It is obvious that the parameter κ in the classifier and iterative cycles *T* are the factors that determine the improvement of classification accuracy.

According to the law of large numbers in probability theory, while N→∞, the next-round sample number will tend to Nε. Assume $R^{N}\to \left\{0,1\right\}$ denotes the map from query state to their labels, there are two conditional probabilities of classification error, i.e.,

(21) $$\begin{array}{cc} \; & p\left(x_{i}\mapsto y_{i}=0|x_{i}\mapsto y_{i}=1\right)=p\left(x_{i}\mapsto y_{i}=1|x_{i}\mapsto y_{i}=0\right)\\ \; & =1-\kappa_{i}, \end{array}$$

where a↦b denotes that *a* is labeled as *b*. Few research achievements at present pay attention to the post-processing of classifier. We present a soft method to boost the state fidelity in the quantum classifier. After the first cycle of classification, one method is to classify the same quantum state |Φ〉 (or $|\Phi^{g}\rangle$) after adjusted the weights of $|\varphi_{j}\rangle$ to implement the second cycle. In this method, the weights of lower fidelity states will be raised. The success probability of the same sample will be classified above threshold after several rounds of classification. Aside from this method, another method is that the sample states under classification threshold can be constructed as a new state and can again be proceeded. After every cycle of implementing classification, the number of error data will be reduced rapidly.

The base classifier coefficients integrate error rate and sample weight distribution status, which together act on the base classifier, making it more accurate than relying solely on error rate to evaluate the classifier. The base classifier coefficients, combined with double error measurement, optimize the update process of sample weights, making it focus on difficult to distinguish samples while increasing the diversity of the classifier.

## 4. Experiments

According to the fuzzy number due to the transmission deviation in the quantum channel, given that the probabilities $P_{0}$ and $P_{1}$ are based on the circuit in Figure 4, the measurement result may be gained in the following form

(22) $$|\Phi \rangle =\frac{1}{2\sqrt{N}}\sum_{n=1}^{N}\left|n\rangle (|0\rangle \right|\Phi_{\hat{x}+x_{n}}\rangle +|1\rangle |\Phi_{\hat{x}-x_{n}}\rangle )|y_{n}\rangle ,$$

where $|\Phi_{\hat{x}\pm x_{n}}\rangle =|\Phi_{\hat{x}}\rangle \pm |\Phi_{x_{n}}\rangle$ for $\hat{x}$ is the test data and each $x_{n}$ is training data, respectively. Furthermore, $|\Phi_{\hat{x}}\rangle$ and $|\Phi_{x_{n}}\rangle$ are prepared states involved with test state and training state, and *w* is distribution weight. Therefore, the measurement probability of the class label $y_{n}$ in state 0 is

(23) $$P_{0}=\frac{1}{2\sqrt{N}}\sum_{y_{n}=0}|\hat{x}+x_{n}{|}^{2}=1-\frac{1}{2\sqrt{N}}\sum_{y_{n}=0}{\left|\hat{x}-x_{n}\right|}^{2}.$$

The proposed classifier to distinguish the different labels resorts to the distance between test state and training state, hence we investigate experimentally the performance affected by different angular amplitudes. The probability to achieve success is

(24) $$P_{s}=\frac{1}{4N}\sum_{n=1}^{N}{\left|\hat{x}+x_{n}\right|}^{2}.$$

### 4.1. Algorithm

According to the proposed algorithm, the classical data are initially encoded into the quantum states using rotations parameterized by the input data. The trained circuit can then be used to predict the labels of the test data. The subset based on transformed qubits are then measured to gain the output in the form of expected value which are decoded into the class labels. Therefore, we present the following Algorithm 1 based on the above process.    
**Algorithm 1:** Quantum classifier with respect to quantum encoding**Prepare:** Sample set *X*, unlabeled test point $\hat{x}$ and quantum classifier circuit QC.**Input**: graph G=(V,E), adjacent matrix Γ1. **for** $x_{i}\in X$1≤j≤N, **do**encode $x_{i}$ into $|x_{i}\rangle$ with quantum phase encoder.2. Applying *H* to entangle the sample states with Γ,so that two-level graph state coupling graph is formed.3. Resort to the circuit QC,**for** 1≤j≤M **do**obtain *M* classes of weak quantum classifiers $h_{j}$.4. Computing the distances between $|\hat{x}\rangle$ and $|x_{i}\rangle$**Output**: The label *y* that $|\hat{x}\rangle$ belongs to.

Presently, there are several major quantum software frameworks, including Google’s qsim, IBM’s Qiskit Aer, Xanadu’s PennyLane and Classiq’s Quantum Algorithm Design platform. IBM recently released the Qiskit Aer, so users have the chance to complete circuit design, simulation, practical computation and so on [38].

This presents great possibilities for researchers to test and verify their theories and algorithms in the quantum field. Qiskit Aer launched by IBM is a high-performance simulator of open-source framework for quantum computers, which provides a highly adjustable noisy model and its corresponding tools for operating quantum applications [39]. Its functionality allows users to execute programs of online accessible quantum emulators, that can further promote the research and development of quantum computer algorithm and benchmark test. Qiskit machine learning is an application module built on the existing functions of Qiskit, which expands machine learning applications by combining quantum computing and machine learning technology. It has added basic computational building modules, including quantum core and quantum neural network, which are designed for different applications such as classification or regression. In this experimental environment, the Qiskit Aer simulator is taken as the tool to test and evaluate the results under quantum computation scenarios. Here, there are 80 gates from a set of 12 single-qubit quantum logic gates that are allowed in this experiment. Firstly, the designed classifier in this experiment was implemented with the quantum processor and tested on Iris dataset [40]. The Iris data set is the oldest dataset, which first appeared in the 1936 by the famous British statistician and biologist Ronald Fisher, and was used to introduce linear discriminant analysis. These data are originally stored in the well-known University of California, Irvine (UCI) dataset repository. It consists of three physical parameters of flowers, i.e., Versicolor, Setosa, and Virginia. The numerical parameters included in the dataset are Iris Setosa, Iris Versicour, and Iris Virginia. Furthermore, four features sepal length, sepal width, petal length and petal width are included in the dataset. We divide the Iris dataset into a training set and a testing set, typically using 70% of the data as the training set and 30% as the testing set. Each category collected 50 samples, so this dataset contains a total of 150 instances. For building a binary classification, the two first classes and two features (sepal width and petal length) of the Iris dataset are chosen in the experiment. The features at the initialization phase are normalized into every superposition graph state. Within the capabilities of the device, we consider two features of two samples from the Iris dataset for the implementation. We take two Iris samples, 42 and 91, which correspond to two training vectors $x_{1}=\left(0.002,0.985\right),y_{1}=0$ and $x_{2}$ = (0.812, 0.584), $y_{2}$ = 1 in training dataset $S_{1}=\left\{\left(x_{1},y_{1}\right),\left(x_{2},y_{2}\right)\right\}$, respectively. Furthermore, the two input vectors of class 0 are denoted as ${\hat{x}}_{1}=\left(0.485,0.875\right)$ and ${\hat{x}}_{2}=\left(0.053,0.999\right)$ in the Iris dataset. As a result, the probability pair $\left(P_{0},P_{1}\right)$ of the two input data are (0.536,0.464) and (0.612,0.388), respectively, with success experimental probability $P_{s}=0.673$. Correspondingly, the probability pair of simulation prediction is (0.629,0.371) with $P_{s}=0.835$. We can see from the results that the success probability of simulation results is higher than that of experimental results. It is mainly due to the lack of error correction, so the classifier can be considered as a weak quantum classifier $L_{qw}$.

### 4.2. Boosted Classification Algorithm and Comparison

According to the previous description, the apparent error influences the performance of classification. To boost the precision, we consider adjusting the weights between the classifiers $h_{1},h_{2},\ldots ,h_{M}$ coupling the subgraphs of network graph *G*. By running the weak quantum learning algorithm $L_{qw}$ on various distributions over domain *X*, the weights of these training state $|x_{n}\rangle$ will be boosted to a stronger classifier. In term of the property of quantum entanglement with each of the vertices, we consider an *N*-site lattice in the whole graph with a qubit attached to each site. The sample state $|x_{n}\rangle$ is expanded into space $S_{N}$, so a state vector $|{\tilde{x}}_{n}\rangle$ can be generated. Similarly, the corresponding ancilla state vector $|{\tilde{\varphi }}_{n}\rangle$ also can be obtained. At the beginning, the equal weight corresponding to each query state is initialized. To the states with wrong labels, the corresponding weight will be increased. To the states with wrong labels, the corresponding weight will be increased. For reducing their weights, the wrong-label states will be highlighted and prepared again, so that it will be endowed with a new attribute distribution. After *T* cycles, the low fidelity will be boosted ultimately. Firstly, the last-round weight of $M^{\prime }$ error query states is normalized to a probability distribution $w_{t,i}/\sum_{i=1}^{M^{\prime }}w_{t,i}$. Then, for each new set of query states, we calculate their updated error rate in terms of their new weights, i.e, $\varepsilon_{t,i}=w_{t-1,i}\varepsilon_{t-1,i}$. Based on the obtained new error rate, the next round of weight can be endowed with $w_{t+1,i}=w_{t,i}\alpha^{1-\varepsilon_{i}}$ for $\alpha_{t}=\varepsilon_{i}/\left(1-\varepsilon_{i}\right)$. In fact, $\varepsilon_{i}$ is the minimum error rate, then $1-\varepsilon_{i}$ is maximum accuracy. At last, we will gain a boosted quantum classifier $h({\hat{x^{\prime }}}_{i})$ in terms of their mixed state $\rho_{C}^{t}$ to obtain precision vector $\vec{p^{t}}$, that if it satisfies $2\sum_{t=1}^{T}\log\alpha_{t}h_{t}\left({\hat{x^{\prime }}}_{i}\right)\leq \sum_{t=1}^{T}\alpha_{t}$, the test state is labeled 1 in set *Y*, otherwise 0. The above process can be described as follows in Algorithm 2.
**Algorithm 2:** Boosted quantum classifier with *T* cycles**Input**: Quantum training dataset $(|x_{i}\rangle ,y_{i})\in X\times Y$;weak learning algorithm $L_{qw}$;integer *T* of iterative cycle;form sample state vector $|\tilde{x}\rangle$ and ancilla vector $|\tilde{\varphi }\rangle$**Initialize** the weight of graph$w_{1,n}=D_{1}\left(n\right)=\frac{1}{\sqrt{2N}}$ for n=1,⋯,N, and ${\hat{w}}_{1,n}$**for** 1≤t≤T, **do**1. Construct cluster states $|{\vec{h}}^{\left(t\right)}\rangle_{C}$ and $|{\vec{h}}_{a}^{\left(t\right)}\rangle_{C}$2. Compute mixed state $\rho_{C}^{t}$ to obtain $\vec{p^{t}}$3. Apply $L_{qw}$ to provide $\vec{p^{t}}$, return $h_{t}:X\to \left[0,1\right]$.4. Obtain the error $\varepsilon_{t}$ of $h_{t}$.5. Update weights vector ${\vec{w}}_{t+1}$**Output**: the H(x) of graph state |G〉.

To depict the capability of the boosted method of iterative cycles with quantum circuits, we perform the following experiments based on the Iris and Skin classifications. Because of the imbalanced and small dataset in Iris, we increase the number of samples of the minority class on another dataset, i.e., Skin dataset. On this dataset, the 245,057 instances with two classes are implemented, which is also originally derived from the machine learning repository of UCS. It is based on the Euclidean distance, and the attributes of R(red) and B(blue) colors among RGB colors of the pixel in this dataset are considered. Firstly, the prepared qubit state will be transformed as a series of unitary rotation parameterized with trainable weights and entanglements in Figure 4. Correspondingly, the algorithm is used in neural network graph similar to Figure 5 in terms of the unitary rotation gates parameterized by trainable parameters. The classifiers in network subgraphs can be depicted as a graphical representation of tensors, in which the vertices are entangled by their vertices according to adjacent matrix and encoded with five-qubits. In every iterative cycle, the tensor is a weight vector which aims to represent the updated vector corresponding to the gate parameters in the graph. The three classes of results (experimental result, simulation result and theoretical result) are derived from three cycles of the above described algorithm, as are listed in Table 1.

Table 1 shows the results for small scale that includes the number of quantum qubit and CNOT gate involved in the quantum circuit in Figure 4 and Figure 5. It is obvious that the simulation result and theoretical result are always higher than the experimental result in the table.

After boosting the algorithm, we compare the results to several classical methods. Furthermore, we also present the experimental results of comparing the proposed boosting algorithm against prior works with implementation details over metrics. Table 2 and Table 3 further show the experimental results for the baseline determined with the classical and quantum machine learning models of the boosting algorithm on the Iris dataset and Skin dataset. In the tables, KNN algorithm over classical model is usually looked on as the main baseline with an accuracy of approximately 94% in Table 2, and 93% in Table 3, respectively. For the sake of evaluating the effectiveness of the quantum boosting (Qboosting) classification algorithm, the experimental results are illustrated to compare with previous quantum KNN algorithm [6]. In this algorithm, the distance between samples, such as Euclidean distance or Manhattan distance, are usually calculated for measurement. In practical applications, some other classical classification algorithms such as decision trees and SVM, etc., can also be used based on the two datasets. The implementation can be achieved by using the machine learning libraries in Python.

Due to limitations of the accessible quantum hardware, only two training vectors with two features for the classifier are involved in the two experiments, so that the classifiers are kept as simple as possible. Hence, after the test set was selected randomly from the union of the test and training data over Iris and Skin datasets, the results of an experiment can bbe shown in the provided tables. Furthermore, quantum KNN and the proposed quantum boosting algorithms with three cycles over quantum model are applied to the datasets, which can be achieved by available five-qubits quantum computers. Furthermore, we also consider the performance of classical learning algorithms as the comparison. We can see from the baseline, that the minority class is difficult to separate from the majority class. Owing to the instances in the Iris dataset being relatively small, we can observe that the result between quantum model and classical model is not very obvious in Table 2. In contrast, the advantage of quantum classifiers lies in the large amount of data due to the entanglement property. The test error between the implement quantum algorithms will be enlargened while the instances increase.

**Figure 9:**
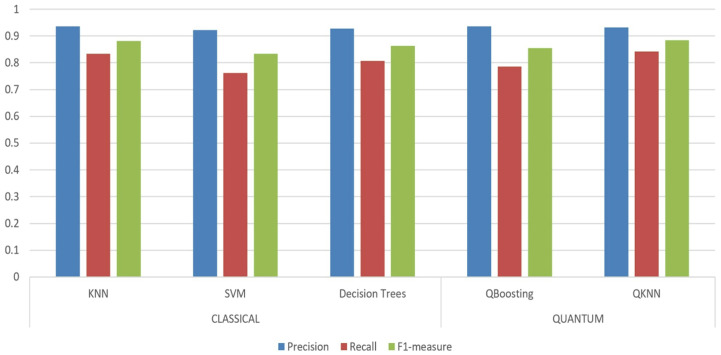
Metrics comparison of quantum boosting (Qboosting) and QKNN with classical KNN, SVM and Decision Trees via use of the UCI Skin dataset.

Furthermore, the stronger classifier of boosting algorithms achieves better performance than quantum KNN for precision. However, the additional running time will be consumed from the process complexity. However, the 0.0084s running time of the proposed quantum boosting algorithm is longer than 0.0025s of QKNN algorithm. Owing to the amplitude encoding to encode the sample data, we standardize the features with zero mean and unit variance to normalize the sample vectors. An adequate choice of training vectors strongly influences the probability of classification success, and the errors are shown in the final column in the two tables. As following, the metrics comparison of several algorithms is visually displayed in the histogram Figure 9.

### 4.3. Running Time Analysis

For comparison, a classical classifier algorithm of obtaining classification results generally takes time polynomial on vector number and space dimension. Generally, the total run time of the classical algorithm is correspondingly $O(N^{2}\left(N+\mathrm{poly}\left(m\right)/{\left(1-\varepsilon \right)}^{2}\right))$. If any test data vectors $\tilde{x}$ are to be classified as one of the result 1 or 0, the quantum query state correspondingly will be taken as a normalized quantum vector $|\vec{\hat{x}}\rangle$ in the proposed algorithm. It is efficient to process big data in high-dimensional spaces with quantum graph state, so realizing learning tasks with quantum machine bears advantages over the classical one. Since the quantum state involves the *m*-dimensional feature space, the total O(logmNM) run time is implemented in both training based on the feature attribute and classification steps in one round of classification. The inner product evaluation in classical classification will achieve *O*(poly${\left(MN\right))/\left(1-\varepsilon \right)}^{2}$ with the distributed uneven weights [41]. However, the same operation inquantum mechanism can generally achieve the running time of O(M).

To encode the classical data into $\log_{2}N$ qubit entangled graph state, $O(\log_{2}N)$ steps are estimated efficiently. In an ideal environment of quantum circuit, the complexity of the *M* query states based on the feature space will achieve run times of O(MlogN/(1−ε)). On the other hand, the dot products of all quantum vertex vectors are estimated to the same degree of accuracy. Hence, it takes the evaluation of single dot product of the classification in the higher-dimensional space as the following representation

(25) $${\vec{x}}_{n}\cdot {\vec{x}}_{k}=|{\vec{x}}_{n}\left|\right|{\vec{x}}_{k}|\langle {\vec{x}}_{n}|{\vec{x}}_{k}\rangle ,\;\;\;n,k=1,2,\ldots ,N$$

in this classification algorithm. Its process takes time O(logN) by calculating inner products and *N* quantum sample states run times O(log(mN)), i.e., O(logN) in running the proposed algorithm. Furthermore, the involved distances and inner products of quantum states in *N*-dimensional vector spaces, by combining *M* test states takes time O(logMN) [4]. Therefore, according to the dependency on components distribution of sample state vector $\vec{x}$, the running time of described quantum algorithm with *T* cycles can reach up to O(TN(N+logm/(1−ε))).

From the results, it is shown that the ability of quantum computers to manipulate high-dimensional vectors will be more efficient to polynomial kernel especially in entanglement quantum system.

## 5. Conclusions

Applying effective means to represent classical data with a quantum data logical structure in machine learning can open up opportunities for enhancing various existing implementation methods. Efficient quantum state storage is crucial in quantum computing. The big data storage of graphic structure bears certain advantages over linear or tree structures. Multi-partite entanglement state in quantum computing has its own advantage in physical property. In terms of the structural characteristics of quantum training state and test state in machine learning, we build a map between so-called two-level nested graph state to feature space that is further expanded to network, so that it builds the bridge between classifier and the research on neural network. Large-scale query data encoded into this quantum form can be classified into two labels with swap-test classification. For further boosting the fidelity, an iterative classification method is proposed by adjusting the weight of each round of error quantum states so that a strong classifier can be obtained. All quantum query states will be classified with a probability close to 1 after several cycles, i.e., the classification fidelity is rapidly improved. Based on the Iris dataset and Skin dataset, the proposed quantum algorithm is implemented with hardware in this paper. Experimental results show that the graph method can enhance the performance in benchmark strengthening.

## Figures and Tables

**Table 1:** By implementing five-qubits circuit, the classification of three cycles for experimental result, simulation result and theoretical result of the input vector from Iris dataset and Skin dataset.

Dataset	Qubits	Cycle	Experimental (%)	Simulation (%)
Iris	5	1	83.51	87.92
		2	96.20	97.58
		3	98.37	98.86
Skin	5	1	67.33	73.54
		2	76.46	79.59
		3	83.12	84.85

**Table 2:** Classification comparison with classical and quantum models, in terms of test average accuracy for 5 runs. The dataset taken is the Iris dataset, and quantum KNN is the baseline in the experiment. The results for KNN, SVM and decision trees in classical model.

Dataset	Model	Method	Precision (%)	Recall (%)	F1-Measure (%)	Qubit Error
Iris	Classical Model	KNN	94.12	94.06	94.09	
		SVM	93.54	93.26	93.40	
		Decision Trees	93.82	94.01	93.91	
	Quantum Model	QBoosting	95.34	96.06	95.70	0.0183
		QKNN	94.67	95.56	95.11	0.0192

**Table 3:** Classification comparison with classical and quantum models over Skin dataset in terms of test average accuracy for 5 runs.

Dataset	Model	Method	Precision (%)	Recall (%)	F1-Measure (%)	Qubit Error
Skin	Classical Model	KNN	93.54	83.41	88.19	
		SVM	92.23	76.13	83.41	
		Decision Trees	92.78	81.62	86.30	
	Quantum Model	QBoosting	93.57	78.57	85.42	0.0327
		QKNN	93.21	84.13	88.43	0.0438

## Data Availability

The data in the supporting paper can be provided by the authors based on reasonable requirements.

## References

[1] Nielsen M., Aand Chuang I.S. (2000). Quantum Computation and Quantum Information.

[2] Cirac J.I., Zoller P. (2000). Scalable quantum computer with ions in an array of microtraps. Nature.

[3] Sasaki M., Carlini A. (2002). Quantum learning and universal quantum matching machine. Phys. Rev. A.

[4] Rebentrost P., Mohseni M., Lloyd S. (2014). Quantum support vector machine for big data classification. Phys. Rev. Lett..

[5] Lu S., Braunstein S.L. (2014). Quantum decision tree classifier. Quantum Inf. Process..

[6] Hu W. (2018). Comparison of Two Quantum Nearest Neighbor Classifiers on IBM’s Quantum Simulator. Nat. Sci..

[7] Biamonte J., Wittek P., Pancotti N., Rebentrost P., Wiebe N., Lloyd S. (2017). Quantum machine learning. Nature.

[8] Blank C., Park D.K., Rhee J.K.K., Petruccione F. (2020). Quantum classifier with tailored quantum kernel. npj Quantum Inf..

[9] Du Y., Hsieh M.H., Liu T., Tao D. (2021). A Grover-search based quantum learning scheme for classification. New J. Phys..

[10] Liao H., Convy I., Huggins W.J., Whaley K.B. (2021). Robust in practice: Adversarial attacks on quantum machine learning. Phys. Rev. A.

[11] Li Y., Meng Y., Luo Y. (2021). Quantum Classifier with Entangled Subgraph States. Int. J. Theor. Phys..

[12] Zhou N.R., Zhang T.F., Xie X.W., Wu J.Y. (2023). Hybrid quantum Cclassical generative adversarial networks for image generation via learning discrete distribution. Signal Process. Image Commun..

[13] Briegel H.J., Raussendorf R. (2001). A One-Way Quantum Computer. Phys. Rev. Lett..

[14] Mandel O., Greiner M., Widera A., Rom T., Hnsch T.W., Bloch I. (2003). Controlled collisions for multi-particle entanglement of optically trapped atoms. Nature.

[15] Raussendorf R., Browne D.E., Briegel H.J. (2003). Measurement-based quantum computation using cluster states. Phys. Rev. A.

[16] Dür Aschauer W.H., Briegel H.J. (2003). Multiparticle Entanglement Purification for Graph States. Phys. Rev. Lett..

[17] Walther P., Pan J.W., Aspelmeyer M., Ursin R., Gasparoni S., Zeilinger A. (2004). De Broglie wavelength of a non-local four-photon state. Nature.

[18] Hein M., Eisert J., Briegel H.J. (2004). Multi-party entanglement in graph states. Phys. Rev. A.

[19] Clark S.R., Alves C.M., Jaksch D. (2005). Efficient generation of graph states for quantum computation. New J. Phys..

[20] Aschauer H., Dur W., Briegel H.J. (2005). Multiparticle entanglement purification for two-colorable graph states. Phys. Rev. A.

[21] Hu D., Tang W., Zhao M., Chen Q., Yu S., Oh C.H. (2008). Graphical Nonbinary Quantum Error-Correcting Codes. Phys. Rev. A.

[22] Park D.K., Petruccione F., Rhee J.K.K. (2019). Circuit-based quantum random access memory for classical data. Sci. Rep..

[23] Schuld M., Petruccione F. (2018). Supervised Learning with Quantum Computers.

[24] Schuld M., Sinayskiy I., Petruccione F. (2015). An introduction to quantum machine learning. Contemp. Phys..

[25] Esma A., Gilles B., Seebastien G. (2006). Machine learning in a quantum world. Advances in Artificial Intelligence.

[26] Wittek P. (2014). Quantum Machine Learning: What Quantum Computing Means to Data Mining.

[27] Pudenz K.L., Lidar D.A. (2013). Quantum adiabatic machine learning Quantum. Quant. Inf. Proc..

[28] Turkpence D., Akncß T.Ç., Şeker S. (2019). A steady state quantum classifier. Phys. Lett. A.

[29] Schuld M., Fingerhuth M., Petruccione F. (2017). Implementing a distance-based classifier with a quantum interference circuit. EPL (Europhys. Lett.).

[30] Danielsen L.E. (2005). On self-dual quantum codes, graphs, and Boolean functions. arXiv.

[31] Li Y., Ji C.L., Xu M.T. (2014). Nested Quantum Error Correction Codes via Subgraphs. Int. J. Theor. Phys..

[32] Gottesman D. (1996). Class of quantum error-correcting codes saturating the quantum Hamming bound. Phys. Rev. A.

[33] Calderbank A.R., Rains E.M., Shor P.W., Sloane N.J. (1997). Quantum error correction and orthogonal geometry. Phys. Rev. Lett..

[34] Calderbank A.R., Shor P. (1996). Good quantum error-correcting codes exist. Phys. Rev. A.

[35] Cong I., Choi S., Lukin M.D. (2019). Quantum convolutional neural networks. Nat. Phys..

[36] Huang H.Y., Broughton M., Mohseni M., Babbush R., Boixo S., Neven H., McClean J.R. (2021). Power of data in quantum machine learning. Nat. Commun..

[37] Schuld M., Bradler K., Israel R., Su D., Gupt B. (2020). Measuring the similarity of graphs with a gaussian boson sampler. Phys. Rev. A.

[38] IBM Quantum Experience. www.research.ibm.com/quantum.

[39] Qiskit. https://qiskit.org/.

[40] Fisher P.A. (1936). The use of multiple measurements in taxonomic problems. Ann. Eugen..

[41] Aaronson S. (2009). Quantum Machine Learning Algorithms: Read the Fine Print. arXiv.

